# Comparison of the Efficacy Between Articaine and Lignocaine in Simultaneous Bilateral Orthodontic Maxillary Premolar Extractions: A Split-Mouth Comparative Study

**DOI:** 10.7759/cureus.50078

**Published:** 2023-12-06

**Authors:** Sai Krishna, Rajprakash Bhaskaran, Santhosh P Kumar, Murugesan Krishnan

**Affiliations:** 1 Department of Oral and Maxillofacial Surgery, Saveetha Dental College and Hospitals, Saveetha Institute of Medical and Technical Sciences, Saveetha University, Chennai, IND

**Keywords:** innovative technique, novel therapies, local anesthesia, pain, therapeutic extraction, orthodontic extraction, lignocaine hydrochloride, articaine hydrochloride

## Abstract

Introduction

Local anesthesia plays a crucial role in ensuring patient comfort during orthodontic extractions. Among the various local anesthetic agents commonly used in the field of oral surgery are articaine and lidocaine, which differ in their duration of action and pain effectiveness (pain control) during surgical procedures. This article aimed to analyze the characteristics of 2% lignocaine with 1:80000 adrenaline and 4% articaine with 1:100000 adrenaline regarding duration of action and pain control in patients undergoing bilateral orthodontic maxillary premolar extractions.

Materials and methods

A split-mouth comparative study was conducted at Saveetha Dental College and Hospitals, Chennai, for which 50 patients of age less than 30 years and who required bilateral orthodontic premolar extractions were selected. Approximately 4% articaine hydrochloride solution was administered on one side, and 2% lignocaine hydrochloride was administered on the contralateral side. Palatal infiltration was not given in the articaine group. The degree of extraction difficulty was similar in both groups, with no discernible variation. In each patient, the duration of anesthesia and pain control were assessed. The IBM Statistical Package for Social Sciences (SPSS version 24.0, IBM SPSS Statistics for Windows, Armonk, NY) was used to perform the student's paired t-test for detecting the difference in outcome parameters between the two groups.

Results

Upon comparing both groups, it was concluded that the articaine group had a longer mean anesthetic duration of action of 217 minutes, whereas for the lignocaine group, it was 169 minutes, and greater pain reduction was present with the articaine group. The articaine group exhibited less pain (superior pain control) with a mean visual analogue scale (VAS) score of 1.07 compared to that of the lignocaine group with a mean VAS score of 1.53 during orthodontic premolar extractions. Both the results were statistically significant (P=0.001).

Conclusion

This split-mouth comparative study concludes that articaine is a more effective local anesthetic in terms of duration of action and pain reduction than that of lignocaine, and it can be used as a local anesthetic of choice for orthodontic maxillary premolar extractions.

## Introduction

Orthodontic extractions or therapeutic extractions are usually indicated in patients undergoing orthodontic treatment where the tooth-to-arch length ratio is not adequate. Usually, in most of the cases, maxillary and mandibular first premolars are indicated for extractions. Orthodontic extractions are minor oral surgical procedures performed under local anesthesia, and the efficacy of the local anesthetic plays a crucial role in the success of the treatment [1].

Local anesthetics are essential in preventing or reducing the perception of pain by blocking nerve impulses in the area where they are applied. Two widely used local anesthetics in dentistry are articaine and lidocaine, which have different characteristics that can affect their effectiveness in terms of duration of action and pain management during orthodontic extractions. Both articaine and lignocaine belong to the same group of anesthetics (i.e., the amide group but structurally they differ). Articaine contains both amides and an ester ring, whereas lignocaine lacks an ester ring in its structure [2].

The presence of the ester group allows articaine to undergo rapid metabolism in both the blood and tissues, leading to a longer duration of action compared to other amide-type local anesthetics like lignocaine. Apart from metabolism, the ester group also has a role in protein binding. As there is increased protein binding, there will be an increased duration of action. This prolonged effect makes articaine particularly useful in procedures that require a more extended period of anesthesia, such as complex orthodontic extractions. On the other hand, lignocaine undergoes a slower metabolism, resulting in a shorter duration of action, and may necessitate additional anesthetic injections for more complex or lengthy orthodontic procedures. Pain control is one more important factor as it contributes to patient comfort and overall satisfaction. Dense attachment of the palatal mucosa to the underlying periosteum renders infiltration over the palatal mucosa a painful procedure [3]. 

According to Lekholm and Zarb, the type of density of the bone in the anterior region of the maxilla is more of cancellous with thin cortical bone. Articaine has higher penetration and diffusibility. It has been shown that articaine has a faster onset of action, allowing for quicker anesthesia administration and reduced discomfort during the injection process. Apart from this property, the extended duration of action of articaine ensures that patients remain comfortable throughout the procedure, experiencing minimal pain. These findings suggest that articaine may provide superior pain control compared to lidocaine. However, it is essential to note that individual patient factors, such as age, gender, overall health, and previous experiences with local anesthetics, can influence the response to these medications [3]. Some patients may exhibit variations in their response to different anesthetics, and considering these factors is crucial in selecting the most appropriate local anesthetic for orthodontic extractions. A comprehensive understanding of their characteristics is essential for oral and maxillofacial surgeons and dental professionals [4] as it will ultimately enhance the quality of life for their patients [5].

This study aimed to evaluate the efficacy of articaine over lignocaine in terms of duration of action and effectiveness in terms of pain reduction during orthodontic maxillary premolar extraction procedures.

## Materials and methods

Study design and setting

This prospective comparative study was conducted at Saveetha Dental College and Hospital, Chennai, over three months. Ethical approval was obtained from the institution (IHEC/SDC/OMFS-2204/23/152), and written informed consent was taken from all the participants before the study. 

Inclusion criteria

A total of 50 participants of age ranging from 15-30 years requiring bilateral orthodontic extractions were enrolled in the study. The participants who were categorized under the American Society of Anesthesiologists - I (ASA-I) and presented cooperative behavior were included in the study.

Exclusion criteria

Patients who are medically compromised like those with diabetes and hypertension, pregnant females, those with anxiety, smokers, alcoholics, those with a history of allergy to anesthetic medication, and those having inflammation at the site of injection were excluded from the study.

Protocol

The extraction sites were randomly allocated either to receive articaine or lignocaine groups using a computer-based randomization sequence. Randomization was performed by an individual not involved in the data collection. The independent observer covered 25 cartridges, each containing 1.8 mL of 2% lignocaine hydrochloride, and 25 cartridges, each containing 1.7 mL of 4% articaine hydrochloride with colored tape in violet and red, respectively, to prevent identification. The staff members who were in charge of dispensing the masked cartridges provided the randomization codes; however, they were not involved in the delivery of the drugs or evaluation of the results. The double-blind characteristic of the experiment was ensured because neither the patient nor the surgeon knew the details of the type of local anesthesia delivered.

Procedure

**Figure 1:**
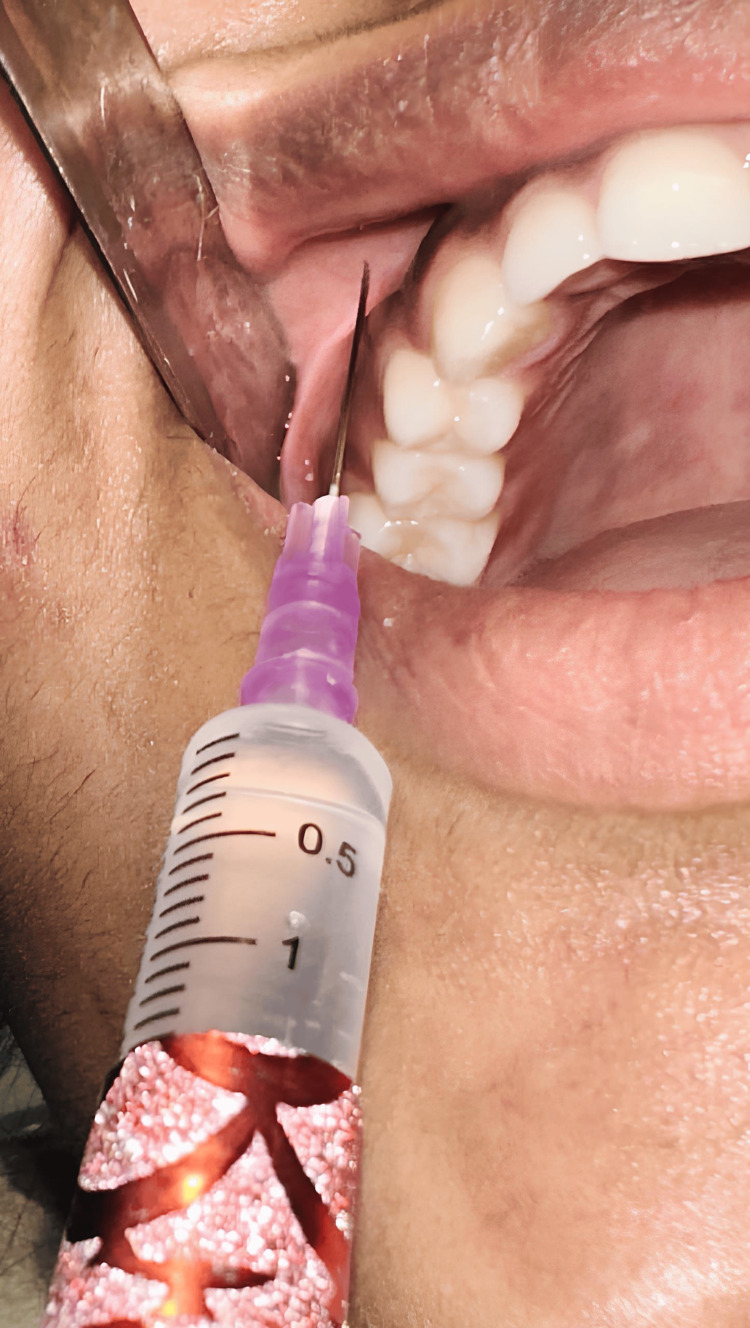
Orthodontic premolar extraction performed using articaine

**Figure 2:**
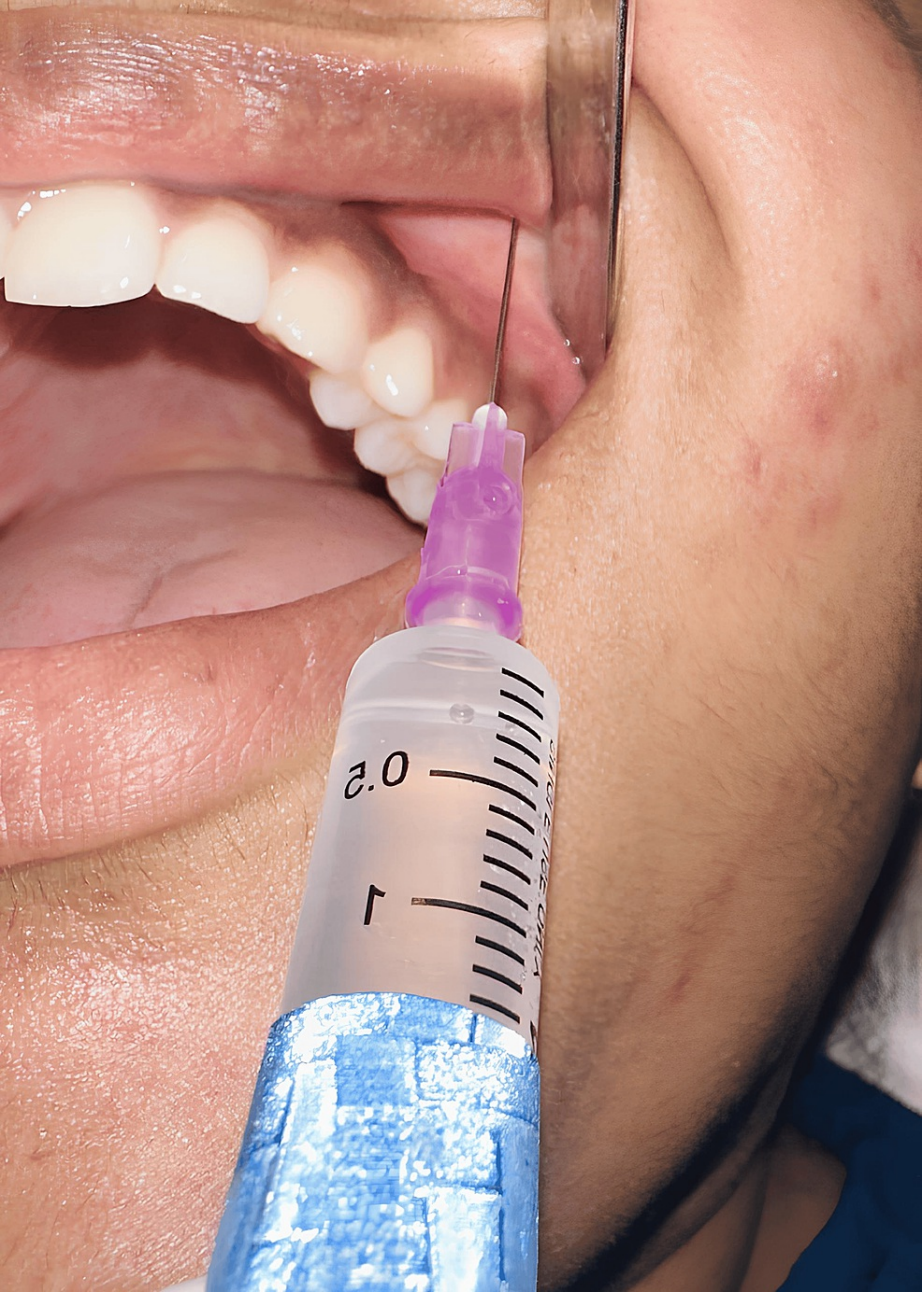
Orthodontic premolar extraction performed using lignocaine

Each participant underwent bilateral orthodontic extractions, with one extraction site receiving articaine (Figure 1) and the contralateral site (Figure 2) receiving lignocaine. On the day of the procedure, after obtaining baseline vital signs, a syringe with a 27-G needle was used to deliver a conventional maxillary infiltration injection. Needle insertion was done in the vestibular regions of the maxillary premolar, and 1.2 mL of solution was deposited in both groups. No palatal infiltration was given in the articaine group, whereas 0.5 mL of solution was given in the lignocaine group. All the extractions were conducted by a single oral and maxillofacial surgeon. Electric pulp testing was conducted as an objective test for every one, two, four, six, and eight minutes to evaluate the onset of pulpal anesthesia. Patients were asked subjectively to confirm the action of local anesthesia. Once after attaining the pulpal anesthesia, the duration of anesthesia was calculated until the anesthesia wore off. After successful anesthesia, under sterile aseptic conditions, extraction was conducted with standard forceps, and pressure was applied with a gauze pack. Patients were advised to bite the gauze for 30 minutes. Analgesics (paracetamol and aceclofenac combination) were prescribed postoperatively for three days. 

Outcome measures

Duration of action was measured from the time of attaining pulpal anesthesia (objective symptom) using the electric pulp tester until the patient attained reversal of sensation (subjective symptom) and was calculated in minutes. Pain effectiveness (pain control) is the patient's perception of pain during the extraction and was assessed through patient-reported pain scores using a standardized 5-point visual analog scale (VAS).

Data collection and analysis

The information was collected and input into a 2013 Microsoft Excel spreadsheet. Statistical Product and Service Solutions (SPSS version 24.0; IBM SPSS Statistics for Windows, Armonk, NY) was used to perform the student's paired t-test for detecting the difference in outcome parameters between the two groups. A P value less than 0.05 was considered statistically significant.

## Results

The age and gender distribution of our study participants is depicted in Table 1. Among 50 participants, 40 were male patients, 10 were female patients, 40 patients were in the age range of 15-20 years, and 10 patients were in the age range of 20-25 years. Two groups were included in the study: group 1 was the articaine group, and group 2 was the lignocaine group. Among these two groups, the parameters evaluated were the duration of action and pain perception of the patient during the extraction procedure.

**Table 1:** Demographic variables of the study participants

Gender	15-20 years	20-25 years	Total
Male	35	5	40
Female	5	5	10
Total	40	10	50

A longer duration of action was seen with articaine (group 1), with a mean duration of 217.47 minutes, compared to that of lignocaine (group 2), with a mean of 169.00 minutes. Patient perception of pain during the extraction procedure was measured using a five-point VAS scale, and the data showed that the mean pain score was low with group 1 (articaine, i.e., 1.07) compared to that of group 2 (lignocaine, i.e., 1.53). Thus group 1 (articaine) exhibited a longer duration of anesthesia, as well as effective pain reduction than group 2 (lignocaine), and the results were statistically significant (P<0.001) (Table 2).

**Table 2:** Comparison of outcome parameters among the two study groups Group 1 - articaine anesthesia, Group 2 - lignocaine anesthesia, ** Statistically significant - paired t-test (P<0.001**)

Parameter	Groups	Number	Mean	Standard deviation	P value
Duration of action	1	50	217.47	4.291	0.001**
	2	50	169.00	6.403	-
Pain scores	1	50	1.07	0.258	0.001**
	2	50	1.53	0.640	-

**Figure 3:**
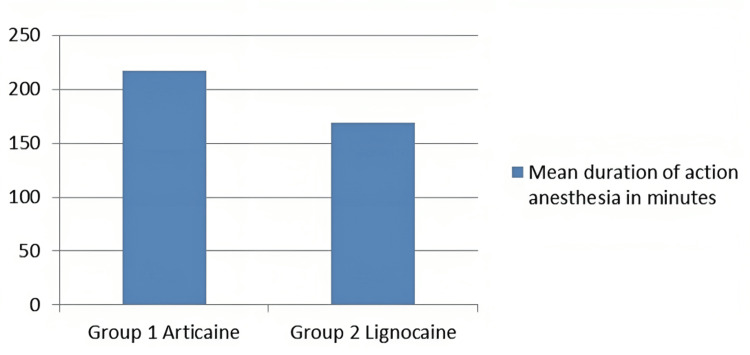
Mean duration of action among the two study groups in minutes Group 1 (articaine), Group 2 (lignocaine)

Figure 3 depicts that the articaine group demonstrated a longer mean duration of action (217.47 minutes) compared to that of the lignocaine group (169.00 minutes).

**Figure 4:**
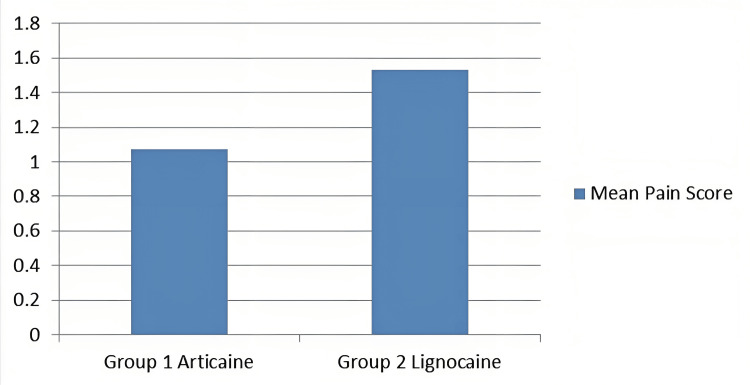
Mean pain scores among the two study groups (VAS scores) Group 1 (articaine), Group 2 (lignocaine)

Figure 4 depicts that the articaine group exhibited less pain (superior pain control) mean VAS score of 1.07 compared to that of the lignocaine group with a mean VAS score of 1.53 during orthodontic premolar extractions.

## Discussion

All the participants involved in the study were of an age less than 30 years and graded as ASA I grade [6]. Patients without any underlying systemic comorbidities such as diabetes mellitus were included in the study as its prevalence is higher in dental patients [7]. This was done to ensure the patient's safety and to prevent adverse effects that might be caused by the administration of articaine.

The present study compared the duration of action, and pain effectiveness (pain control) during extraction between articaine and lidocaine for orthodontic extractions through a split-mouth design. The study's findings revealed that articaine demonstrated a prolonged duration of action compared to lidocaine. This aligns with existing literature that suggests articaine's unique chemical structure, incorporating a thiophene ring, may contribute to its extended anesthetic effect [6,7]. The results correspond with several previous studies that have highlighted the sustained numbing effect of articaine, making it a favorable choice in dental procedures that demand longer anesthesia, such as orthodontic extractions [6-8].

Regarding pain effectiveness, our study's outcomes indicated that articaine yielded superior pain control compared to lidocaine during orthodontic extractions. This can be attributed to articaine's increased tissue penetration because of its higher lipid solubility, facilitating a more profound and reliable block of nerve impulses [8]. Consequently, patients who received articaine experienced less discomfort and pain during the procedure, contributing to an improved overall patient experience. Patients' perception of pain during the extraction was assessed using a visual analog scale. The method most frequently employed in many dental operations is the local infiltration technique [9]. The success rate of maxillary infiltration is higher compared to that of infiltrations in the mandibular arch because of cortical thickness and marrow space. When used properly, it offers both the operator and patients outstanding comfort and safety, similar types of studies were even performed for different difficulty levels of impacted third molars [10]. The pharmacokinetics and level of toxicity of local anesthetics must be taken into consideration. Today, lidocaine serves as the benchmark against which all new local anesthetics are measured [11].

In this study, the duration of action of anesthesia was measured from the time of onset of anesthesia until the anesthesia wore off. The onset of anesthesia was measured using the electric pulp testing, which was an objective symptom, and wear-off in anesthesia was confirmed by questioning the patient (subjective symptom). Pulpal anesthesia can be achieved in 60 minutes with 2% lignocaine in a vasoconstrictor ratio of 1:10,00,00. Because of the aromatic ring's replacement, articaine is 1.5 times more powerful than lignocaine [12]. Electrical stimulation can be used to assess pulpal anesthesia in healthy, asymptomatic teeth. The results of Dreven et al. were used as the foundation for the criteria for pulpal anesthesia [13]. At a reading of 80, if the patient doesn't respond, that suggests profound pulpal anesthesia. An electric pulp tester reading of less than 80 caused discomfort in teeth that were otherwise asymptomatic, according to Certosimo et al. [14]. To achieve pulpal anesthesia, electric pulp testing must be done on critical teeth. The pulp tester reading of 80 was also cited by Haas et al. [15] as the standard for pulpal anesthesia in the aforementioned research. Vishal et al. [16] and Bansal et al. [17] also observed similar findings, and the degree to which an anesthetic binds to proteins determines how long the effect lasts.

Several studies have shown similar results with articaine having a higher duration of action compared to that of lignocaine, and it was attributed to the role of thiophene ring in the articaine group [12,14-16]. According to the studies by Potocnik et al. [18] and Borchard et al. [19] articaine has more potency in blocking A fibers than of lignocaine. Hassan et al. in their studies have shown that the onset of anesthesia was more rapid with the articaine group than that of lignocaine as the solubility and penetration of articaine were higher [20]. Kakroudi et al. compared articaine with other local anesthetic agents, and it was shown that articaine has superior properties in terms of rapid onset of anesthesia and longevity of anesthesia [21].

Age is another factor that might play a role in the patient's perception of pain. In a study conducted by Somuri et al., it was shown that children below 20 years of age never experienced pain during the surgical procedure when articaine was used as an infiltration agent [22]. Articaine is a hybrid molecule with both ester and amide groups in its structure, which aids in superior properties compared to that of lignocaine and palatal injection, which is painful, can be avoided with articaine local anesthetic thereby yielding to less discomfort and better cooperation from the patient.

The future scope of our research would include employing larger sample sizes, investigating patient-related variables, and possibly extending the investigation to a broader spectrum of dental procedures.

Limitations of the study

Though our study design consisting of bilateral simultaneous orthodontic extraction procedures controls for inter-individual variability, less sample size may impact the generalizability of the results. Additionally, factors like patient anxiety and pain threshold, which can influence pain perception, were not extensively explored.

## Conclusions

Articaine demonstrated a longer duration of action and superior pain control compared to lidocaine. The extended duration of action allows for a more comfortable and pain-free experience for patients undergoing orthodontic extractions. The use of articaine may contribute to improved patient satisfaction and reduced anxiety during orthodontic procedures. It can be concluded from the study that articaine with epinephrine is a more effective local anesthetic choice than lignocaine for orthodontic maxillary premolar extractions.
